# Educational interventions to train healthcare professionals in end-of-life communication: a systematic review and meta-analysis

**DOI:** 10.1186/s12909-016-0653-x

**Published:** 2016-04-29

**Authors:** Han-Oh Chung, Simon J. W. Oczkowski, Louise Hanvey, Lawrence Mbuagbaw, John J. You

**Affiliations:** 1280 Main Street West, HSC-2C8, Hamilton, Ontario L8S 4K1 Canada; Hamilton General Hospital, McMaster Clinic 4th floor, Room 434, 237 Barton St East, Hamilton, Ontario L8L2X2 Canada; Canadian Hospice Palliative Care Association, Annex D, Saint-Vincent Hospital, 60 Cambridge Street North, Ottawa, ON K1R 7A5 Canada; St Joseph’s Healthcare, 50 Charlton Avenue East, 3rd Floor Martha Wing, Room H321, Hamilton, Ontario L8N4A6 Canada

**Keywords:** End of life care, Communication, Advance care planning, Advance directives, Communication training, Medical education

## Abstract

**Background:**

Practicing healthcare professionals and graduates exiting training programs are often ill-equipped to facilitate important discussions about end-of-life care with patients and their families. We conducted a systematic review to evaluate the effectiveness of educational interventions aimed at providing healthcare professionals with training in end-of-life communication skills, compared to usual curriculum.

**Methods:**

We searched MEDLINE, Embase, CINAHL, ERIC and the Cochrane Central Register of Controlled Trials from the date of inception to July 2014 for randomized control trials (RCT) and prospective observational studies of educational training interventions to train healthcare professionals in end-of-life communication skills. To be eligible, interventions had to provide communication skills training related to end-of-life decision making; other interventions (e.g. breaking bad news, providing palliation) were excluded. Our primary outcomes were self-efficacy, knowledge and end-of-life communication scores with standardized patient encounters. Sufficiently similar studies were pooled in a meta-analysis. The quality of evidence was assessed using GRADE.

**Results:**

Of 5727 candidate articles, 20 studies (6 RCTs, 14 Observational) were included in this review. Compared to usual teaching, educational interventions to train healthcare professionals in end-of-life communication skills were associated with greater self-efficacy (8 studies, standardized mean difference [SMD] 0.57;95 % confidence interval [CI] 0.40–0.75; *P* < 0.001; very low quality evidence), more knowledge (4 studies, SMD 0.76;95 % CI 0.40–1.12; *p* < 0.001; low quality evidence), and improvements in communication scores (8 studies, SMD 0.69; 95 % CI 0.41–0.96; *p* < 0.001; very low quality evidence). There was insufficient evidence to determine whether these educational interventions affect patient-level outcomes.

**Conclusion:**

Very low to low quality evidence suggests that end-of-life communication training may improve healthcare professionals’ self-efficacy, knowledge, and EoL communication scores compared to usual teaching. Further studies comparing two active educational interventions are recommended with a continued focus on contextually relevant high-level outcomes.

**Trial registration:**

PROSPERO CRD42014012913

**Electronic supplementary material:**

The online version of this article (doi:10.1186/s12909-016-0653-x) contains supplementary material, which is available to authorized users.

## Background

Advances in medical care and the aging population have highlighted the need for good end-of-life (EoL) communication and decision-making, in order to ensure that invasive medical treatments are not administered to patients who would prefer less aggressive forms of care at the end-of-life [1]. Unfortunately, health care providers (HCPs) often fail to engage patients in EoL discussions and to document patient wishes in the medical chart [1, 2]. This puts many patients at risk of having unwanted aggressive and potentially futile medical care during their last days of life, which is associated with worsened patient and caregiver quality of life and psychological burden [3].

An important strategy for improving the quality of EoL discussions is to improve EoL communication skills amongst HCPs [4]. The educational need for this skill has been well described for both trainee and practitioners alike: medical graduates currently are entering practice ill-prepared to discuss the important EoL issues with patients and families [5–7]. In a multicenter Canadian survey, internal medicine residents at five universities identified that EoL communication skills were a high learning priority [8] as resident physicians are often responsible for facilitating EoL discussions with hospitalized patients in academic centers [7, 9, 10]. This need persists in even practicing HCPs such as physicians and nursing staff who continue to have discomfort in facilitating EoL discussions [11–15]. There are numerous EoL communication skills training programs described in literature, however the cumulative evidence on the impact of such an educational intervention remain unclear. Therefore, we conducted a systematic review to evaluate the effectiveness of educational interventions to train HCPs in EoL communication skills compared to usual teaching (i.e. standard curriculum). The effectiveness was measured based on the Kirkpatrick training evaluation model (Reaction, Learning, Behaviour and Results) [16] which was represented by outcomes of self-efficacy, knowledge, communication skills and patient-level effects.

## Methods

### Protocol and registration

The protocol for the complete review is available in the PROSPERO database at: http://www.crd.york.ac.uk/PROSPERO/display_record.asp?ID=CRD42014012913.

### Eligibility criteria

Studies were eligible for our systematic review if they included adult patients over age 18 years, healthcare providers, or trainees, and if they evaluated a communication tool to assist adult patients in EoL decision-making, in comparison to a control group. Our definition of a communication tool included traditional decision aids in any format (paper, video, computer, etc.), and other structured approaches to help with decision-making, including organized meeting plans, reminders to complete advance directives (AD) or educational interventions for patients or healthcare providers. Interventions designed solely for information-sharing (e.g. breaking bad news, providing emotional support) were excluded, because although such interventions may affect EoL decision-making, it is not their sole or explicit purpose to do so. We included randomized controlled trials (RCTs) and prospective observational studies with a control group (including cohort studies and uncontrolled before-after studies in which participants acted as their own control). We restricted the review to studies published in peer-reviewed journals in the English language (See Additional file 1).

Eligible studies were then divided into a subgroup of studies of educational interventions for health care providers, and a subgroup of studies used as clinical intervention. In this paper, we specifically review only the studies of educational interventions directed to health care providers, whether trainees in a health professional training program (e.g. medical or nursing student, post-graduate training), or practicing providers receiving continuing medical education. Reviews of the patient directed end-of-life communication tools will be analyzed and reported elsewhere.

### Outcome measures

Based Kirkpatrick model of evaluation *Reaction* measures the learners’ value they perceive in the educational intervention. *Learning* measures improvements in their knowledge, *Behaviour* measures their capability applied in context, and *Result* measures the impact the training on the target outcome – in this case, patient level outcomes [16].

With this framework, primary outcome measures were:

1. Self-efficacy (participant’s confidence or estimate of their ability to perform a task) [17].
2. Knowledge test scores on EoL communication and decision-making.
3. Communication scores using a standardized checklist during a standardized patient encounter.

Secondary outcome measured patient-level outcomes such as completion of AD, health care utilization, patient satisfaction with EoL planning, and patient assessment of clinician communication skills.

### Search strategy

A comprehensive search was performed for papers available for search from database inception to July 2014 from Medline (1946 – July 2014), Excerpta Medica database (EMBASE 1980 – July 2014), Cumulative Index to Nursing and Allied Health Literature (CINAHL 1982 – July 2014), Cochrane Database of Controlled Clinical Trials (2005 – July 2014) and Education Resources Information Center (ERIC 1966 – July 2014). Searches were conducted using terms related to EoL decision-making and communication, including: *“communication,” “decision-making,” “end-of-life”* and “*cardiopulmonary resuscitation*.”. A snowball technique was used to hand search references for additional papers for review. This review is a subset of a larger systematic review on EoL decision-making interventions. Only those studies relevant to medical education are reviewed here. Reviews of communication interventions evaluated in the clinical setting will be analyzed and reported elsewhere.

### Study selection

Title and abstracts were screened for relevance independently and in duplicate by two reviewers (SO, HC). Articles that passed initial screening by either reviewer underwent full-text review independently and in duplicate by the same two reviewers. Standardized, piloted eligibility forms were used for both title and abstract screening, and for full-text review. Disagreements about study eligibility were resolved through consensus discussion or resolved by a third reviewer (JY) in the case of ongoing disagreement. Kappa statistics were calculated to assess the inter-rater reliability of title and abstract screening and full-text review [18].

### Data collection and data items

Data extraction was done using standardized, piloted, online forms by two reviewers (HC, SO), including publication information, study dates and population characteristics, interventions, outcomes, and study methods required to assess the risk of bias. We contacted study authors to obtain missing information relevant to outcomes or risk of bias.

### Study quality and risk of bias of individual studies

Educational study quality and risk of bias were assessed in duplicate by two reviewers using the MERSQI (Medical Education Research Study Quality Instrument) Scale and Newcastle-Ottawa Scale Education (NOS-E) [19, 20]. The two instruments were used as they assess different aspects of quality and risk of bias acting in a complementary fashion. We considered a score above the sample median MERSQI score (12.5) and NOS-E score (2.5) as the threshold for high methodological quality as described in other literature [20].

The risk of bias for RCTs was additionally assessed using the Cochrane risk of bias tool which includes assessments of random sequence generation, allocation concealment, blinding of participants and personnel, incomplete outcome data, and selective reporting. Each domain was assessed independently by both reviewers and reported as being at ‘high,’ ‘low,’ or ‘uncertain’ risk of bias. Studies were considered to be at overall ‘high’ risk of bias if judged to be at ‘high’ risk of bias at any domain; ‘uncertain’ risk of bias if judged to be at uncertain risk of bias in any one domain, with no domains at high risk of bias; and at overall ‘low’ risk of bias, if not judged to be at ‘high’ or ‘uncertain’ risk of bias in any domains [21]. All risk of bias assessment was judged at the outcome level.

### Publication bias

Publication bias was assessed using visual inspection of funnel plots, where sufficient numbers of studies existed to permit interpretation [22].

### Data synthesis, summary measures and sensitivity analysis

Reviewers assessed studies for clinical heterogeneity by investigating study populations, interventions, and comparisons before considering whether to pool data. We assessed statistical heterogeneity for each of the outcomes of interest using the I^2^ statistic, with values greater than 50 % indicating significant heterogeneity [23].

We used Review Manager 5.3 software to calculate pooled estimates of effects using all relevant studies employing the generic inverse variance method. A random-effects model was used to pool weighted outcomes of standardized mean differences (SMD). The magnitude of effect was interpreted in accordance with the Cohen effect size classification (small 0.2–0.5, moderate 0.5–8, large >0.8) [24]. Estimates for standard deviation for change scores were calculated when not reported or obtainable from study authors [25]. To see if the methodologic quality would materially affect our findings, sensitivity analyses were conducted post-hoc by restricting pooling of studies to those of higher methodologic quality such as RCTs only, or high MERSQI or NOS-E scores.

### Ratings of quality of evidence

We used the Grading of Recommendations Assessment, Development, and Evaluation (GRADE) approach to assess the quality of evidence for each outcome of interest. To rate the quality of evidence, the GRADE approach considers, for each outcome of interest, risk of bias within each study; risk of bias across studies (e.g. publication bias); imprecision of results; inconsistency of results; and indirectness of the evidence [26]. Summary of finding tables were generated using GradePRO software [27].

## Results

### Study selection

**Fig. 1:**
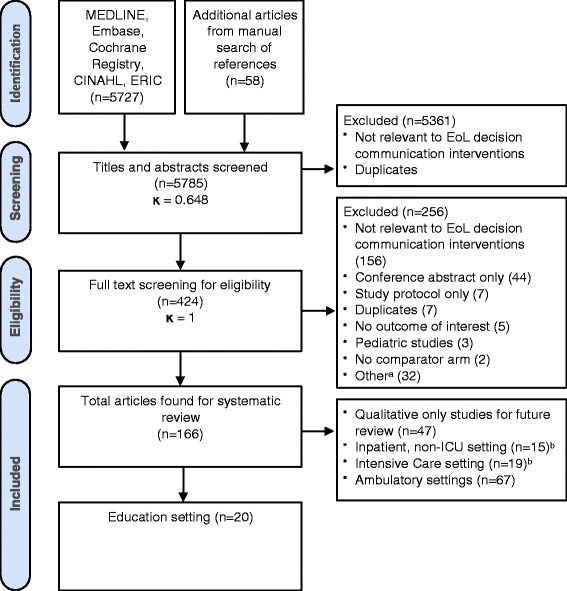
Flow diagram of study screening and eligibility. ^a^Marked ‘other’ due to unclear documentation whether study excluded due to duplication or non-relevance. ^b^One educational study overlapped with the inpatient studies and two overlapped with the ICU studies

Initial database searches retrieved 5727 articles. After exclusion of duplicate references, conference abstracts, and title and abstract screening, 424 articles were selected for full-text review (κ = 0.65, 95 % CI [0.60, 0.70]). A total of 166 articles were found to be eligible for inclusion after full-text review and additional manual reference screening. Of these, 20 were studies of educational interventions and were reviewed in this paper (Fig. 1).

### Study characteristics

#### Study setting & populations

Most of the studies were completed in the USA (80 %), and 17 (90 %) were aimed toward medical trainees (14 postgraduate level, 3 undergraduate medical school), one to postgraduate medical trainees and nurse practitioners in acute care programs, and two studies were open to all acute care HCP (Table 1).

**Table 1:** Study characteristics

Study ID	Design	Intervention group, n	Control group, n	Learner description	Country	Intervention	Comparator	Outcome
Alexander 2006 [38]	Cohort	37	19	Ambulatory care rotator residents in Duke University Medical Centre	USA	The two-day retreat involving small-group lecture/ discussion, audio-visual materials, recordings of actual physician–patient encounters, and scripted “trigger tapes.” Learners practiced through supervised role-play.	No intervention	Communication Behaviour
Back 2007 [39]	Pre/Post	100 Participants		Oncology fellows from 62 different institutions who applied for the specialized workshop	USA	The Oncotalk curriculum involved a 4 day retreat, taught in small groups of 5 participants and 1 faculty facilitator. The curriculum was organized around 5 simulated patients. Learning activities included overviews, skills practice sessions, and reflective discussions.	Pre-intervention	Communication Behaviour
Bristowe 2014 [30]	Pre/Post	16 Participants		Hemodialysis Nurses/Physicians	UK	Communication workshop with following sessions: fact session; patient and carer experience session; professional and personal experience session; communication, role-playing and feedback	No intervention	Self-Efficacy
Clayton 2012 [31]	Pre/Post	21 Participants		Residents at large tertiary-referral teaching-hospital in Sydney, Australia, voluntary participation	Australia	Three 1 hour, onsite teaching sessions and a follow-up telephone call, spread out over 4 weeks. Sessions included interactive presentation of evidence-based strategies for conversations with patients expected to die within days/weeks and their caregivers and practice with standardized patients and feedback from an expert facilitator. Follow-up phone call one week after final session reinforced and extended learning, and offered further support and feedback. Written and audio take-home learning materials provided.	Pre-intervention	Self-Efficacy
								Communication Behaviour
Fischer 2007 [35]	Pre/Post	29 Participants		Primary care internal medicine residents, voluntary participation	USA	Workshop included a brief lecture, group discussions, role-playing, and videotape review. Topics included included breaking bad news, advance care planning, ethics.	Pre-intervention	Knowledge
Furman 2006 [5]	Pre/Post	8 Participants		Internal Medicine and medicine-paediatric Residents at Louisville Veterans Affairs Medical Center	USA	One morning report session consisting of both didactic training and three-person role-played discussion.	Pre-intervention	Patient AD Document
Green 2011 [34]	RCT	60	56	Second year medical student in Penn State College of Medicine	USA	Students help patients create advance directives using a multimedia decision-aid which helps patients clarify their values, explain end-of-life conditions, help users choose and communicate with their surrogate decision makers and translate their wishes and goals.	Students using Standard AD	Patient Satisfaction with EoL and EoL Care planning
								Student Knowledge
Greenberg 1993 [28]	RCT	46	47	Medical Students beginning their clerkship	USA	The high-intervention received the same reading as low intervention group as well as a small group seminar on topics of historical development of advance directives, students' experiences with death and dying, contents of a durable power of attorney for health care (DPAHC). Students also viewed a videotape illustrating important aspects of discussing the DPAHC. They were finally assigned to initiate a discussion about advance directives with a patient, family member, or friend.	Provided Self-study readings	Self-Efficacy
								Knowledge
Hales 2008 [4]	Pre/Post	18 Particiants		Multidisciplinary critical care practitioners (MD, RN, SW, RT)	Canada	Workshop participants were assigned to practice groups of three to six members of varying disciplines and institutions. Groups rotated through the six 45-minute stations, enacting scenarios with standardized colleagues and families on topics ranging from the role of the substitute decision maker to approaching families about organ and tissue donation	Pre-intervention	Self-Efficacy
Holloran 1995 [42]	Cohort	Sample size of learners not recorded, outcomes were patient based		Surgical residents rotating through the Surgical ICU (SICU). Outcomes collected for patients who spent more than 30 days in SICU.	USA	Four weekly 60 minute case study discussion groups. Groups led by SICU attending physician and nursing director. Cases designed to force discussion of issues of withholding or withdrawing treatment, eliciting patient and family wishes, incompetent patients and conflict with families	No intervention	Patient AD document
								Health Care Utilization
Lorin 2006 [40]	Cohort	53	53	All 4th year Medical Students rotating through mandatory ICU rotation	USA	Didactic teaching session on ICU communication framework, followed by practice with standardized patients	No intervention	Communication Behaviour
Pekmezaris 2011 [6]	Cohort	77	73	Residents rotating through internal medicine inpatient service in New York	USA	The training was composed of six sessions discussing importance of advance care planing, palliation and life sustaining therapies and interventions. The learners and were involved in role playing with standardized patients and received feedback from expert moderators.	No intervention	Self-Efficacy
Perron 2002 [36]	Pre/Post	9 participants		Residents in Internal Medicine Ward of university affiliated community hospital	Switzer-land	Detailed information was given to all physicians in the department about the meaning of a 'Do not resuscitate' (DNR) order, its ethical dimension, the right of patients to make their own decision , and the concept of medical futility. Ethical aspects addressed only DNR measures and did not cover other measures such as life sustaining treatments.	No intervention	Knowledge
Schell 2013 [32]	Pre/Post	22 Participants		Nephrology Fellows at Duke University and University of Pittsburgh	USA	Session consisting of large group didactic session to highlight the communication skills for breaking bad news and eliciting patient preferences, faculty role-play demonstrating these skills, then the fellows were divided into small groups of five to six members each for skills practice using standardized patients. Fellows had an opportunity to be a practicing fellow or an active observer.	Pre-intervention	Self-Efficacy
Sharma 2014 [37]	RCT	23	28	Residents rotating through internal medicine inpatient service	USA	The intervention group residents completed a multimodality code status discussion (CSD) educational intervention including didactic content, deliberate skills practice and self-study (e.g., online modules and maintenance of a log). In a follow-up intervention, residents received a 2-hour CSD skills “booster” session where they discussed themes from CSD logs, reviewed the CSD framework, and again observed a role play	No intervention	Communication Behaviour
Smith 2013 [33]	Pre/Post	38 Participants		Internal Medicine residents at UCSF	USA	The curriculum consisted of two one-hour lunch conference sessions and six one-hour morning reports at each hospital site, integrated into the regularly scheduled teaching sessions for residents on inpatient rotations. Residents explored challenging patient interactions and to discuss ways for conflict resolution and respond to their own emotional reactions to these scenarios.	No intervention	Self-Efficacy
Szmuilowicz 2010 [29]	RCT	21	28	Second year internal medicine residents at Brigham and Women’s Hospital	USA	One day retreat covering conversations of ‘Breaking Bad News” and “Discussing the Direction of Care”, and skills related to responding to emotions. Every participant had the opportunity to interview a standardized patient and receive feedback from trained faculty at least once during the retreat.	No intervention	Self-Efficacy
								Communication Behaviour
Szmuilowicz 2012 [7]	RCT	19	19	Internal Medicine Residents at Northwestern University	USA	Intervention included a 2 hour seminar discussing advance care planning and framework for EoL conversations, observing a code-status discussion modeled by faculty, and exploration of past experiences. Intervention also included self-study materials, internet communication skills teaching modules and reflective portfolios.	No intervention	Communication Behaviour
Williams 2011 [41]	Pre/Post	24 Participants		All first year internal resident at Thomas Jefferson University Hospital	USA	3-hour workshop began with a review of the evidence behind good communication skills, a discussion of barriers to proper communication, and an in-depth explanation of the SPIKES protocol. Critique and discussion of a communication transcript of actual encounters and 5 minute video highlighting poor communication.	Pre-intervention	Communication Behaviour

Pre/Post refers to pre-intervention/post-intervention (i.e. before-after) studies where participants serve as their own control

#### Study interventions

Review of the instructional design showed the majority of the studies used a combination of didactic lectures (17 studies), small group discussions (16 studies) and role-play with direct observation and feedback (16 studies). A minority of studies included self-study modules (3 studies), video or audio transcript analysis (2 studies), exemplar demonstration (5 studies) and reflective portfolio (1 study). One study used review of a multimedia Advance Directive decision aid with a patient as the educational intervention. Ten studies distributed the learning over more than a day, ten were workshops or tutorials done within a day or less.

### Study quality and risk of bias

Six studies were RCTs, three of which were considered overall ‘high’ risk of bias, and 3 considered to be at ‘unclear’ risk of bias. Of the 14 observational studies, 10 studies were uncontrolled before-after design and 4 studies were double-arm cohort studies with a control group. Mean NOS-E was 3.35 (SD 2.13) out of maximum 6 points. The mean quality of all studies by MERSQI was 11.97 (SD 2.1) out of maximum 18 points. None of the RCTs were rated low overall risk of bias. 11 and 10 studies met the median threshold for high quality by MERSQI or NOS-E criteria respectively (See Additional file 2).

### Synthesis of results

Ratings for the overall quality of evidence and effectiveness of the educational interventions can be seen in the GRADE summary of findings table (See Additional file 3).

1. Self-Efficacy
  Eight studies (2 RCTs [28, 29], 6 Observational [4, 6, 30–33]), including 522 participants found that EoL communication skills training was associated with improved self-efficacy compared to usual training (SMD 0.57; 95 % CI 0.40–0.75; *p* < 0.001; very low quality evidence**)**. There was no evidence of heterogeneity (*I*^2^ = 0 %) (Fig. 2a).

**Fig. 2:**
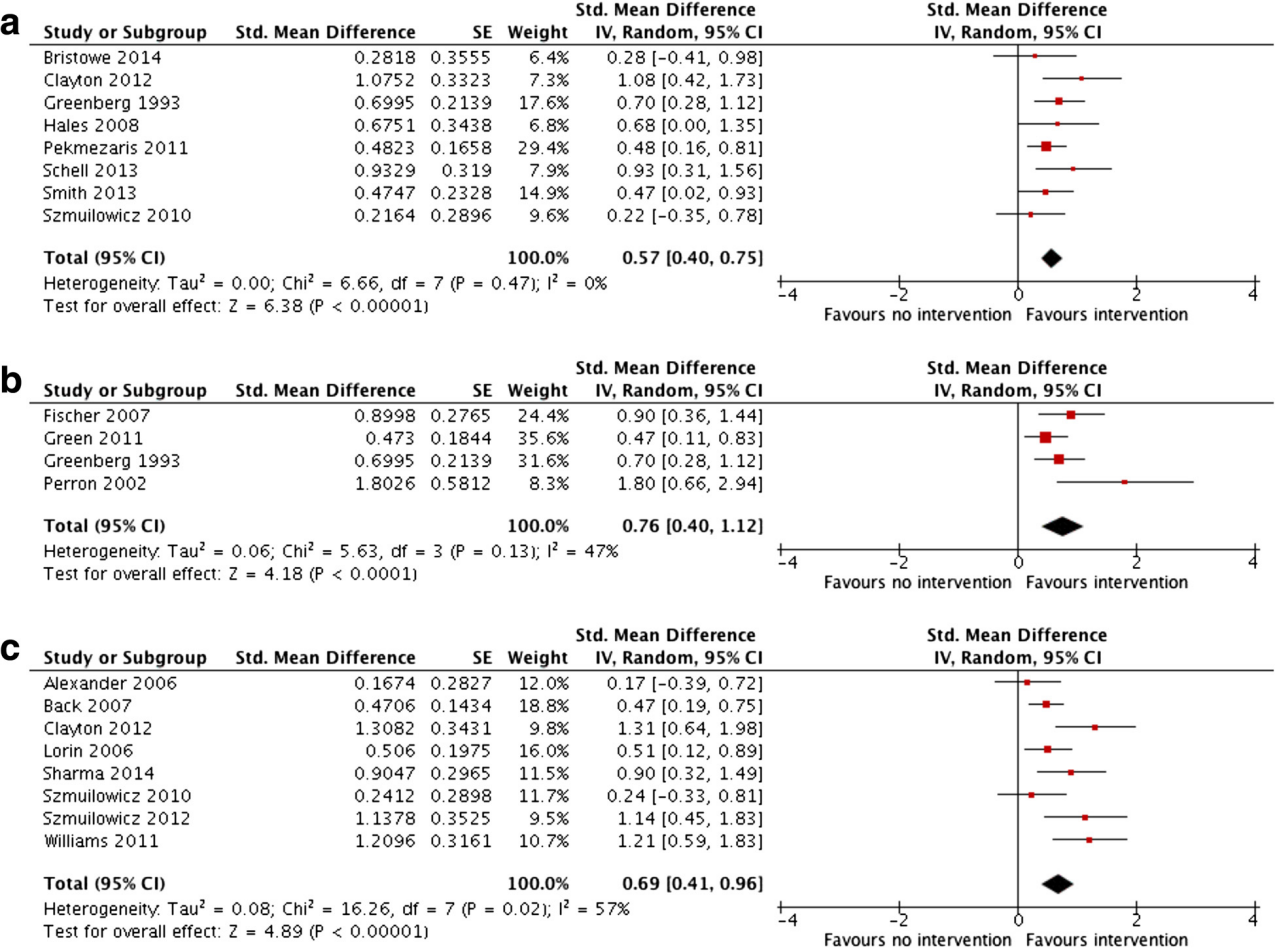
Effect of educational interventions on **a** self-efficacy **b** knowledge and **c** communication scores with standardized patient encounters
2. Knowledge
  Four studies (2 RCTs [28, 34] and 2 Observational [35, 36]), including 290 participants, reported knowledge outcomes. EoL communication skills training was associated with an increase in knowledge scores compared to usual training (SMD 0.76; 95 % CI 0.40–1.12 *p* < 0.001, low quality evidence) with moderate heterogeneity (*I*^2^ = 47 %) (Fig. 2b).
3. Communication score
  Eight studies (3 RCTs [7, 29, 37] and 5 Observational [31, 38–41]), including 590 participants found that EoL communication skills training was associated with improvement in communication scores rated during standardized patient encounters (SMD 0.69; 95 % CI 0.41–0.96; *p* < 0.001; very low quality evidence) with appreciable heterogeneity (*I*^2^ = 57 %). Heterogeneity could not be easily explained by qualitative examination of learner demographics, study quality or instructional design; however in all studies, point estimates of effect were in the direction of benefit for EoL communication skills training (Fig. 2c).
4. Patient outcomes
  There were four studies (2 RCT and 2 Observational) that reported patient-important outcomes. Outcome measures were heterogeneous, precluding pooling of data across studies. Overall, the interventions were neutral to positive: one study found no statistically significant change in the overall proportion of AD completed after a morning educational session [5], whereas another Intensive Care Unit (ICU) based intervention showed a beneficial effect on earlier completion of AD and decreased non-beneficial care in the ICU [42]. One study showed improved patient satisfaction in advance care planning [34], while conversely, another showed no improvement in patient-reported quality of EoL care or quality of communication [43].

### Publication bias

No asymmetry was detected with a visual inspection of the funnel plot for self-efficacy or communication score outcomes, while knowledge outcomes showed some asymmetry. However, due to the limited number of studies, we could not conclusively comment on possible publication bias (See Additional file 4).

### Sensitivity analysis

**Fig. 3:**
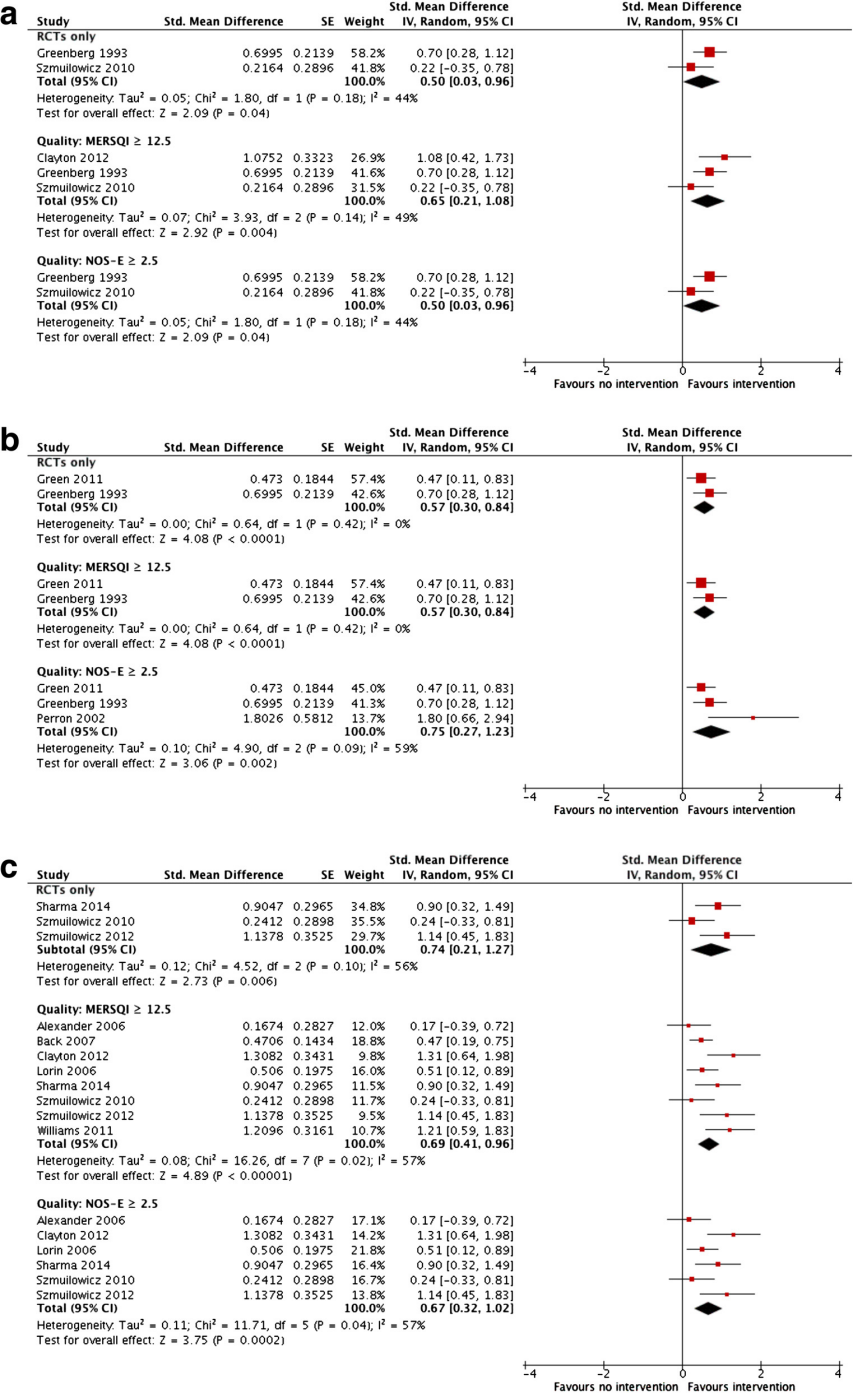
Sensitivity analysis restricting to studies of higher methodologic quality (RCTs only, higher quality MERSQI and NOS-E) for **a** self-efficacy **b** knowledge and **c** communication score

Sensitivity analyses were conducted in which we restricted our pooled analyses to those studies with higher methodological quality: RCTs only, high MERSQI scores only, or high NOS-E scores only. In these sensitivity analyses, the overall direction and magnitude of the effect remained similar after restricting to studies of higher methodological quality (Fig. 3).

## Discussion

In this systematic review, we found very low to low quality evidence from a modest number of studies suggesting that EoL communication training for HCP may improve self-efficacy, knowledge and communication scores compared to no formal training. Our confidence in the effect of these interventions on self-efficacy, knowledge, and communication scores is very low to low primarily because of the high overall risk of bias of individual studies included in the review, as well as imprecision in the pooled results due to small sample sizes. Several studies used uncontrolled pre-post designs, which may overestimate effects due to concurrent co-interventions and maturation effects [44].

Self-efficacy was found to be a common outcome measure as it is easy to measure. However the outcome has important limitations. At best, improving self-efficacy may be beneficial as lack of confidence or negative expectancy may decrease the likelihood that the HCP will voluntarily utilize beneficial communication behaviours [17]. Otherwise, self-assessed performance measures are generally a poor surrogate marker for competence [44]. Earlier studies show physician confidence and actual ability in EoL discussions showed a large disconnect [10, 45]. Similarly, knowledge outcomes do not serve as surrogates to adequate communication skills, as learners may cognitively understand what is important in these discussions, but lack the appropriate skills to carry them out.

We found communication skills outcome the most relevant in capturing the construct of EoL decision-making communication. Our review found evidence of improvement in these measures in the training group, however of very low quality. As well, estimates of effect were quite heterogeneous, which was not easily explained by known study characteristics. There are also potential issues with using a reductionist approach to assessing competence, as expertise may not be adequately captured by binary checklist scores done in these studies [46]. While this way of assessing the outcome measure may be adequate for novice learners, an addition of a subjective global rating may provide a better understanding of their skill in future studies.

We did not find much data on our secondary patient-level outcomes. We found four studies with conflicting effects on the overall benefit of the intervention. This was not unexpected; although patient-level outcomes are an important measure, educational studies rarely have sufficient power or long-term follow-up to detect these high-level outcomes. There are significant confounding variables in between the effect of an educational intervention to finally the effect on patient behavior. This dilution of effect makes it difficult to design the study for adequate power or follow-up length [47]. We must be careful to judge the value of an educational intervention on patient outcomes alone.

### Limitations and strengths

This study is not without limitations. The broad inclusion criteria required to capture a thorough review of the field may have led to some additional heterogeneity and inconsistency in our data, resulting in low quality of evidence according to GRADE, however we suspect that even with more narrow inclusion criteria, the overall quality of the evidence for our outcomes of interest would still be low due to the limited size and quality of studies in this area. We also restricted our search to studies published in the English language. This may limit the applicability of our results to predominantly English-speaking regions.

Other limitations were intrinsic to the available data. We found that the terminology used in the area of EoL communication and decision making is still not well established and is not uniform, which made our literature search difficult. We believe our manual searching of references adequately mitigates this limitation and improves the comprehensiveness of our search, although it is possible that we still missed some relevant studies.

The strength of our review is in the comprehensive literature search with no restrictions with time, inclusion criteria of a broad range of learners, outcomes, and study design; our independent, duplicate screening, eligibility, and quality assessment with rigorous data collection and secondary verification. We also used multiple measures to assess the quality of evidence, using the Cochrane tool, MERSQI or the NOS-E, and conducted sensitivity analyses based on these measures. Finally, we performed comprehensive quality assessments of the totality of evidence using the rigorous GRADE approach.

To our knowledge, this is the only systematic review of educational interventions to train healthcare providers in EoL communication skills that has assessed the quality of evidence using GRADE and conducted meta-analyses to obtain pooled estimates of effect. Other reviews only included a narrative summary of the interventions, with less comprehensive search criteria, and no assessment of study quality [48, 49]. As well, we looked specifically at interventions aimed at improving communication of facilitating and supporting patient decision making on EoL treatment goals, whereas these other reviews looked at a broader skills in palliative care symptom management and breaking bad news.

### Implications

These results generally support the use of structured communication training to improve HCP’s ability to discuss and facilitate EoL decision-making, since they suggest that such training may be effective in improving HCP communication skills. Unfortunately, justification studies such as these that compare against no intervention do not tell us anything aside from the fact that an intervention works [50]. We cannot infer the comparative effectiveness of the different teaching methods, nor can we determine which interventions might be most suitable for a given educational setting or learner population. More studies of higher quality and sound instructional design need to be performed with contextually relevant outcome data and against other active educational comparators.

## Conclusions

In this systematic review, we found consistent, but low-quality evidence that structured communication training, compared to usual curricula, may increase HCP self-efficacy, knowledge, and communication skills for EoL decision-making. While awaiting more robust evidence in this area, educators of health professionals electing to introduce EoL communication skills curricula should continue to design their interventions according to best practice guidelines and base them on a solid theoretical framework.

### Ethics approval and consent to participate

Not Applicable.

### Consent for publication

Not Applicable.

### Availability of data and materials

The datasets supporting the conclusions of this article are included within the article and its additional files. All raw data used in this systematic review are extracted from available published articles. Extracted raw data and Revman analysis files available upon request.

## Additional files

Additional file 1: Search Strategy – Describes our search strategy for various electronic databases. (DOCX 25 kb) Additional file 2: Table S1. Assessment of Study Quality – The rating of individual study quality. (PDF 30 kb) Additional file 3: Table S2. GRADE Summary of Findings for Primary Outcomes – Overall quality of evidence by GRADE criteria. (PDF 47 kb) Additional file 4: Figure S1. Funnel Plot – Funnel plot used to assess for potential publication bias. (PDF 38 kb)

## Abbreviations

AD: advance directives
CI: confidence interval
EoL: end of life
GRADE: grading of recommendations assessment, development, and evaluation
HCP: health care professionals
ICU: intensive care unit
MERSQI: medical educational research study quality instrument
NOS-E: Newcastle Ottawa scale education
RCT: randomized controlled trials
SMD: standardized mean difference
