# Supplementary material for: Educational interventions to train healthcare professionals in end-of-life communication: a systematic review and meta-analysis
Source: BMC Med Educ. 2016 Apr 29;16:131. doi: 10.1186/s12909-016-0653-x (PMC4850701; doi:10.1186/s12909-016-0653-x)
Supplement: Additional file 1: — Search Strategy – Describes our search strategy for various electronic databases. (DOCX 25 kb) [file 12909_2016_653_MOESM1_ESM.docx]

**Electronic search strategies**

Medline (1946-July 2014)

1. exp Hospital Communication Systems/ or exp Communication/ or exp Health Communication/ or exp Interdisciplinary Communication/ or exp Communication Barriers/ or exp Nonverbal Communication/

2. exp Physician-Patient Relations/ed, es, st [Education, Ethics, Standards]

3. *Family/ or exp Family Relations/

4. *Education, Medical, Undergraduate/ or *Clinical Competence/ or *Education, Medical, Graduate/

5. (communic$ adj3 tool$).mp. [mp=title, abstract, original title, name of substance word, subject heading word, keyword heading word, protocol supplementary concept, rare disease supplementary concept, unique identifier]

6. (communic$ adj3 skill$).mp. [mp=title, abstract, original title, name of substance word, subject heading word, keyword heading word, protocol supplementary concept, rare disease supplementary concept, unique identifier]

7. (communic$ adj3 intervention).mp. [mp=title, abstract, original title, name of substance word, subject heading word, keyword heading word, protocol supplementary concept, rare disease supplementary concept, unique identifier]

8. (education$ adj3 tool).mp. [mp=title, abstract, original title, name of substance word, subject heading word, keyword heading word, protocol supplementary concept, rare disease supplementary concept, unique identifier]

9. or/1-8

10. decision making/ or choice behavior/ or "dissent and disputes"/ or negotiating/

11. advance directives/ or living wills/ or contracts/ or informed consent/ or consent forms/ or third-party consent/ or presumed consent/ or resuscitation orders/

12. exp patient care planning/ or advance care planning/ or advance directives/ or living wills/ or critical pathways/ or exp patient-centered care/

13. (deci$ adj3 mak$).mp. [mp=title, abstract, original title, name of substance word, subject heading word, keyword heading word, protocol supplementary concept, rare disease supplementary concept, unique identifier]

14. advance care planning.mp.

15. or/10-14

16. exp Resuscitation/ or resuscitation.mp. or Cardiopulmonary Resuscitation/

17. exp life support care/ or advanced cardiac life support/ or advanced trauma life support care/

18. exp Terminal Care/ or exp Withholding Treatment/ or exp Life Support Care/ or withdrawing life support.mp. or Euthanasia, Passive/

19. Resuscitation Orders/ or dnr.mp.

20. critical illness/ or emergencies/

21. Emergency Service, Hospital/ or Hospital Rapid Response Team/

22. heart arrest.mp. or Heart Arrest/

23. Respiration, Artificial/ or artifical respiration.mp.

1. or/16-23
2. 9 and 15 and 24

Embase (1980-July 2014)

1. exp interpersonal communication/

2. family/ or family assessment/ or family decision making/ or family relation/

3. exp medical education/

4. exp doctor patient relation/

5. (communic$ adj3 tool$).mp.

6. (communic$ adj3 skill$).mp.

7. (communic$ adj3 intervention).mp.

8. (education$ adj3 tool).mp.

9. education program/ or educational intervention.mp.

10. or/1-9

11. exp medical decision making/ or decison making.mp. or exp decision making/

12. exp treatment planning/ or exp patient care planning/ or exp living will/ or care planning.mp. or exp decision making/

13. advance$ directives.mp.

14. advance$ care plan$.mp.

15. (deci$ adj3 mak$).mp.

16. or/11-15

17. exp resuscitation/ or exp emergency treatment/ or exp intensive care/

18. cardiopulmonary resuscitation.mp.

19. exp heart arrest/ or CPR.mp.

20. exp critical illness/

21. life support.mp.

22. Emergency Service, Hospital/ or Hospital Rapid Response Team/

23. Resuscitation Orders/ or dnr.mp.

24. Respiration, Artificial/ or artifical respiration.mp.

25. exp Terminal Care/ or exp Withholding Treatment/ or exp Life Support Care/ or withdrawing life support.mp. or Euthanasia, Passive/

26. or/17-25

27. 10 and 16 and 26

CINAHL (1982-July 2014)

| **Search ID#** | **Search Terms** | **Results** |
| --- | --- | --- |
| S1 | (MH "Communication+") OR "communication" OR (MH "Communication Skills") OR (MH "Communication Skills Training") OR (MH "Communications Media+") OR (MH "Nonverbal Communication+") OR (MH "Communication Protocols+") OR (MH "Communication Barriers") | 379,318 |
| S2 | (MH "Physician-Patient Relations") OR (MH "Professional-Patient Relations+") OR (MH "Nurse-Physician Relations") | 51,346 |
| S3 | (MH "Family+") OR (MH "Family Relations+") | 97,226 |
| S4 | (MH "Education, Medical+") | 14,855 |
| S5 | S1 OR S2 OR S3 OR S4 | 496,220 |
| S6 | (MH "Decision Making+") OR (MH "Decision Making, Patient+") OR (MH "Decision Making, Family") OR (MH "Decision Making, Ethical") OR (MH "Decision Making, Clinical") | 50,433 |
| S7 | (MH "Living Wills") | 691 |
| S8 | (MH "Consent+") | 10,526 |
| S9 | (MH "Resuscitation Orders") | 1,731 |
| S10 | (MH "Advance Care Planning") | 813 |
| S11 | (MH "Advance Directives+") | 5,436 |
| S12 | S6 OR S7 OR S8 OR S9 OR S10 OR S11 | 62,104 |
| S13 | (MH "Resuscitation+") OR (MH "Resuscitation, Cardiopulmonary+") OR (MH "Bystander CPR") | 19,882 |
| S14 | (MH "Advanced Cardiac Life Support+") OR (MH "Life Support Care") | 2,425 |
| S15 | (MH "Terminal Care+") OR "terminal care" OR (MH "Palliative Care") OR (MH "Terminally Ill Patients+") | 36,496 |
| S16 | (MH "Euthanasia, Passive") OR (MH "Treatment Refusal") OR (MH "Treatment Related Pain") OR (MH "Treatment Duration") | 10,486 |
| S17 | (MH "Euthanasia, Passive") | 2,748 |
| S18 | (MH "Heart Arrest+") | 7,719 |
| S19 | (MH "Emergency Service+") | 25,781 |
| S20 | (MH "Respiration, Artificial+") OR (MH "Positive Pressure Ventilation+") OR (MH "Ventilators, Mechanical") | 13,909 |
| S21 | S13 OR S14 OR S15 OR S16 OR S17 OR S18 OR S19 OR S20 | 96,441 |
| S22 | S5 AND S12 AND S21 | 3,492 |
| S23 | S5 AND S12 AND S21 | 1,304 |

Cochrane Database of Clinical Controlled Trials (2005-July 2014)

1 (hospital communication systems or communication or health communication or interdisciplinary communication or communication barriers or nonverbal communication).mp. [mp=title, short title, abstract, full text, keywords, caption text] (1410)

2 Physician-Patient Relations.mp. [mp=title, short title, abstract, full text, keywords, caption text] (17)

3 (Education, Medical, Undergraduate or clinical competence or education, medical graduate).mp. [mp=title, short title, abstract, full text, keywords, caption text] (18)

4 (communic$ adj3 tool$).mp. [mp=title, short title, abstract, full text, keywords, caption text] (19)

5 (communic$ adj3 skill$).mp. [mp=title, short title, abstract, full text, keywords, caption text] (113)

6 (communic$ adj3 intervention).mp. [mp=title, short title, abstract, full text, keywords, caption text] (80)

7 (education$ adj3 tool).mp. [mp=title, short title, abstract, full text, keywords, caption text] (13)

8 (family or family relations).mp. [mp=title, short title, abstract, full text, keywords, caption text] (1736)

9 or/1-8 (2557)

10 (decision making or choice behavior or "dissent and disputes" or negotiating).mp. [mp=title, short title, abstract, full text, keywords, caption text] (802)

11 (advance directives or living wills or contracts or informed consent or consent forms or third-party consent or presumed consent or resuscitation orders).mp. [mp=title, short title, abstract, full text, keywords, caption text] (395)

12 (patient care planning or advance care planning or advance directives or living wills or critical pathways or patient-centered care).mp. [mp=title, short title, abstract, full text, keywords, caption text] (70)

13 (deci$ adj3 mak$).mp. [mp=title, short title, abstract, full text, keywords, caption text] (1041)

14 advance care planning.mp. [mp=title, short title, abstract, full text, keywords, caption text] (8)

15 or/10-14 (1374)

16 (resuscitation or Cardiopulmonary Resuscitation).mp. [mp=title, short title, abstract, full text, keywords, caption text] (245)

17 (life support care or advanced cardiac life support or advanced trauma life support care).mp. [mp=title, short title, abstract, full text, keywords, caption text] (11)

18 (Terminal Care or Withholding Treatment or Life Support Care or withdrawing life support or Euthanasia, Passive).mp. [mp=title, short title, abstract, full text, keywords, caption text] (52)

19 (resuscitation orders or DNR).mp. [mp=title, short title, abstract, full text, keywords, caption text] (4)

20 (critical illness or emergencies).mp. [mp=title, short title, abstract, full text, keywords, caption text] (154)

21 (Emergency Service, Hospital or Hospital Rapid Response Team).mp. [mp=title, short title, abstract, full text, keywords, caption text] (26)

22 Heart Arrest.mp. [mp=title, short title, abstract, full text, keywords, caption text] (18)

23 cardiac arrest.mp. [mp=title, short title, abstract, full text, keywords, caption text] (145)

24 (Respiration, Artificial or artifical respiration).mp. [mp=title, short title, abstract, full text, keywords, caption text] (87)

25 or/16-24 (611)

26 9 and 15 and 25 (61)

ERIC (1966-July 2014)

| **Search ID#** | **Search Terms** |
| --- | --- |
| 1 | exp communication strategies/ or exp communications |
| 2 | exp Physician Patient Relationship/ |
| 3 | (communic$ adj3 tool$).mp. |
| 4 | (communic$ adj3 skill$).mp. |
| 5 | (communic$ adj3 intervention$).mp. |
| 6 | (education$ adj3 tool$).mp. |
| 7 | exp Medical Education/ |
| 8 | or/1-7 |
| 9 | (medical decision making or decision making).mp. or exp decision making/ |
| 10 | exp Medical Services/ or medical treatment.mp. |
| 11 | exp Death/ or exp Decision Making/ or advance directive$.mp. |
| 12 | living will.mp. |
| 13 | care planning.mp. |
| 14 | advance$ directive$.mp. |
| 15 | advance$ care plan$.mp. |
| 16 | (deci$ adj3 mak$).mp. |
| 17 | or/9-16 |
| 18 | end-of-life.mp |
| 19 | (resuscitation or emergency or intensive or critical).mp. |
| 20 | exp first aid/ or CPR.mp. or cardiopulmonary.mp. or arrest.mp. or life support.mp. |
| 21 | DNR.mp. |
| 22 | or/18-21 |
| 23 | 8 and 17 and 22 |
