# Supplementary material for: Educational interventions to train healthcare professionals in end-of-life communication: a systematic review and meta-analysis
Source: BMC Med Educ. 2016 Apr 29;16:131. doi: 10.1186/s12909-016-0653-x (PMC4850701; doi:10.1186/s12909-016-0653-x)
Supplement: Additional file 2: Table S1. — Assessment of Study Quality – The rating of individual study quality. (PDF 30 kb) [file 12909_2016_653_MOESM2_ESM.pdf]

Supplementary Table 1: Assessment of Study Quality

Randomized Control Trials

Study ID	MERSQI	NOS-E	Random Sequence Generation	Allocation concealment	Blinding of participants and personnel	Blinding of outcome assessment	Incomplete Outcome Data	Selective Outcome Reporting	Other
Green 2011	13.5	6	Unclear	Unclear	Low	Low	Low	Low	The authors have potential commercial interest in decision aid used for this study,
Greenberg 1993	12.5	6	Unclear	Unclear	Unclear[1] Low[2]	Low	Low	Low	
Sharma 2014	14.5	6	Unclear	Unclear	Low	Low	Low	Low	
Szmuilowicz 2010	14.5	6	High	Low	Low[3] High[4]	Low	High	Low	
Szmuilowicz 2012	14.5	6	Unclear	Unclear	Low	High	Low	Low	
Curtis 2013	14.5	5	Unclear	Unclear	Low	Low	High	Low	

Observational Studies

Study ID	MERSQI	NOS-E
Alexander 2006	13.5	4
Back 2007	13.5	2
Bristowe 2014	10	0
Clayton 2012	13	2
Fischer 2007	11.5	2
Furman 2006	10	2
Hales 2008	8.5	0
Holloran 1995	11.5	4
Lorin 2006	13.5	6
Pekmezaris 2011	10.5	2
Perron 2002	10	3
Schell 2013	8.5	2
Smith 2013	9	1
Williams 2011	12.5	2

[1] - Unclear Risk for Self-efficacy outcomes
[2] - Low Risk for Knowledge outcomes
[3] - Low risk for Communication Score outcomes
[4] - High risk for Self-efficacy outcomes
