# Supplementary material for: Educational interventions to train healthcare professionals in end-of-life communication: a systematic review and meta-analysis
Source: BMC Med Educ. 2016 Apr 29;16:131. doi: 10.1186/s12909-016-0653-x (PMC4850701; doi:10.1186/s12909-016-0653-x)
Supplement: Additional file 3: Table S2. — GRADE Summary of Findings for Primary Outcomes – Overall quality of evidence by GRADE criteria. (PDF 47 kb) [file 12909_2016_653_MOESM3_ESM.pdf]

Supplementary Table 2: GRADE Summary of Findings for Primary Outcomes

**Outcome**<br>**№ of participants**<br>**(studies)**	**Anticipated absolute effects (95% CI)**<br>**Difference with EoL communication training**	**Difference**	**Quality**
Self-Efficacy assessed with self-assessment<br>№ of participants: 142<br>(2 RCTs)<br>№ of participants: 380<br>(6 observational studies)	The mean self-efficacy in the intervention group was 0.57 standard deviations more (0.40 more to 0.75 more)	SMD **0.57 more**<br>(0.40 more to 0.75 more)	⊕◯◯◯<br>VERY LOW [1,2,3,4,5]
Knowledge assessed with: knowledge test score<br>№ of participants: 214<br>(2 RCTs)<br>№ of participants: 76<br>(2 observational studies)	The mean knowledge in the intervention group was 0.76 standard deviations higher (0.40 higher to 1.12 higher)	SMD **0.76 higher**<br>(0.40 higher to 1.12 higher)	⊕⊕◯◯<br>LOW [3,4,5,6]
Communication skills assessed with standardized patient interaction checklist score<br>№ of participants: 138<br>(3 RCTs)<br>№ of participants: 452<br>(5 observational studies)	The mean communication score in the intervention group was 0.69 standard deviations higher (0.41 higher to 0.96 higher)	SMD **0.69 higher**<br>(0.41 higher to 0.96 higher)	⊕◯◯◯<br>VERY LOW [1,2,3,5]

CI: Confidence interval

GRADE Working Group grades of evidence
High quality: We are very confident that the true effect lies close to that of the estimate of the effect
Moderate quality: We are moderately confident in the effect estimate: The true effect is likely to be close to the estimate of the effect, but there is a possibility that it is substantially different
Low quality: Our confidence in the effect estimate is limited: The true effect may be substantially different from the estimate of the effect
Very low quality: We have very little confidence in the effect estimate: The true effect is likely to be substantially different from the estimate of effect

Justification for grading

1. One of the studies broke randomization
2. One of the two studies show no statistically significant effect
3. Low sample size
4. Individual studies scored poorly on risk of bias assessment
5. Improvements seen in longitudinal before-after studies may be due to intrinsic maturation effects
6. One study showed potential conflict of interest with commercial interest in the studied communication tool
