# Supplementary material for: Educational interventions to train healthcare professionals in end-of-life communication: a systematic review and meta-analysis
Source: BMC Med Educ. 2016 Apr 29;16:131. doi: 10.1186/s12909-016-0653-x (PMC4850701; doi:10.1186/s12909-016-0653-x)
Supplement: Additional file 4: Figure S1. — Funnel Plot – Funnel plot used to assess for potential publication bias. (PDF 38 kb) [file 12909_2016_653_MOESM4_ESM.pdf]

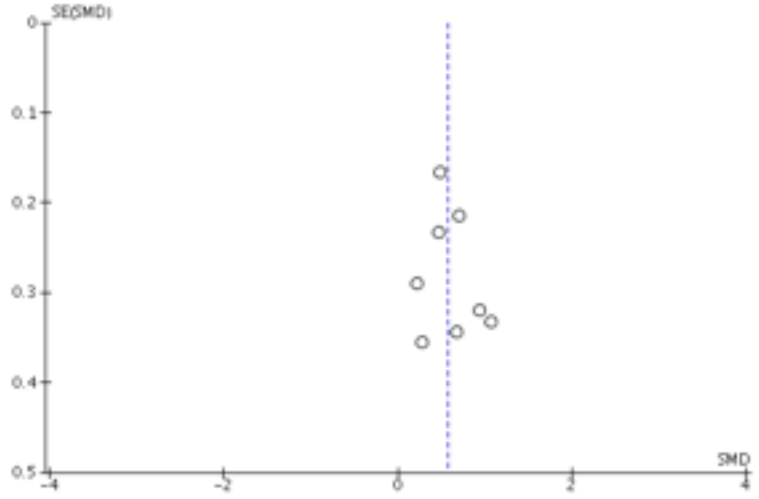

a)

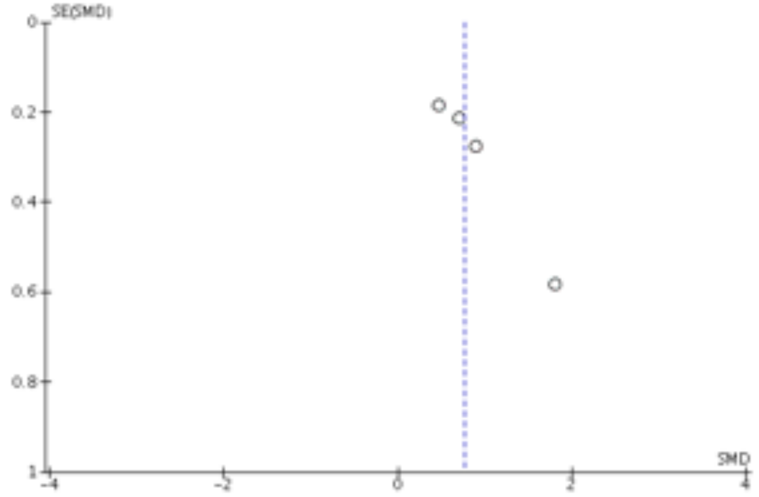

b)

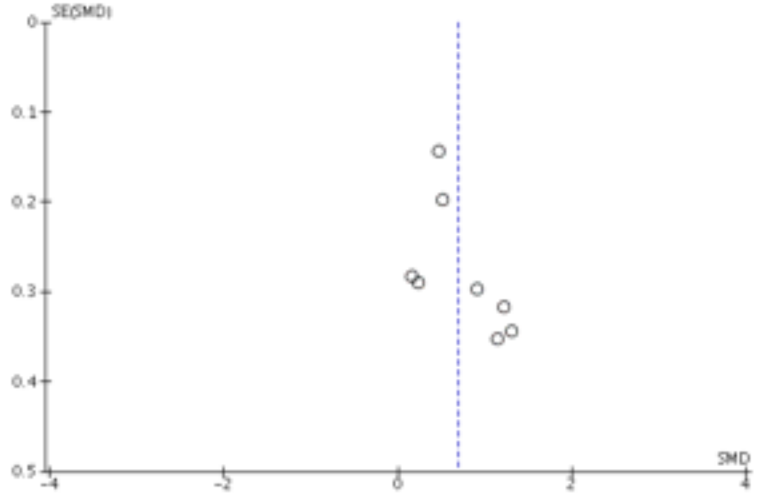

c)

Supplemental Figure. Funnel plot of published studies for a) self-efficacy b) knowledge c) communication score
